# Development of an extended full-time equivalent framework: A workforce metric for the beedi rolling industry in India

**DOI:** 10.18332/tid/203508

**Published:** 2025-06-05

**Authors:** Eshwari Kundangar, Muralidhar M. Kulkarni, Yash Alok, Ambigai Rajendran, Satish Kumar, Praveen Kumar, Priyanka Bantwal, Rohith Bhagawath, Ashwath Naik, Praveen Sinha, Mark Goodchild

**Affiliations:** 1 Department of Community Medicine, Kasturba Medical College, Manipal Academy of Higher Education, Manipal, India; 2 Manipal School of Commerce and Economics, Manipal Academy of Higher Education, Manipal, India; 3 Directorate of Research, Manipal Academy of Higher Education, Manipal, India; 4 Tobacco Control, World Health Organization Country Office, New Delhi, India; 5 World Health Organization, Geneva, Switzerland

**Keywords:** full-time equivalent, beedi rollers, productivity-based model

## Abstract

**INTRODUCTION:**

India’s significant role in the global tobacco industry is underscored by its position as the second largest producer and consumer. Among the various tobacco products, beedis are the most popular in South Asia. These small, thin, hand-rolled cigarettes are wrapped in leaves from native Asian plants and are a traditional form of tobacco consumption in the region. For many women in regions like coastal Karnataka, beedi rolling is a primary occupation and a vital source of income. Labor unions estimate that there are around 7–8 million people in the beedi industry across India, with around 5.5 million registered workers nationwide.

**METHODS:**

A mixed-method approach was initially used to finalize the parameters required for calculating the full-time equivalent (FTE), which comprised expert opinion, scoping review, and focus group discussions among beedi rollers. A 14-item questionnaire was developed, and four experts validated readability, relevance, and clarity in both the local language (Kannada) and English. Content validity was measured using the item-content validity index (I-CVI) and scale content validity index (S-CVI), with a modified Cohen’s kappa value for inter-rater agreement. Subsequently, a cross-sectional design was followed for pilot testing the developed framework for FTE among ten beedi rollers.

**RESULTS:**

The article outlines various frameworks for determining the full-time equivalent (FTE) for beedi workers, broadly categorized into workload-based, productivity-based, and forecasting models. Among these categories, the productivity-based model appears suitable for beedi rollers. Using the extended FTE-index, beedi rolling work was classified as underloaded (≤0.95), normal (0.95–1.04), and overloaded (≥1.05). A pilot study suggests that the proposed FTE framework can be implemented and utilized to assess their workload.

**CONCLUSIONS:**

The study presents a pilot-tested framework to evaluate beedi rollers’ workload and advocates for improved working conditions. Three different models were compared: workload-based, productivity-based, and forecasting. As beedi rollers are paid based on the number of beedis they roll in a week rather than the time it takes, the productivity-based model is best suited for calculating FTE for this occupation.

## INTRODUCTION

Globally, India ranks second in terms of tobacco production and consumption based on the number of adult tobacco users^1^. With an estimated 71.8 million beedi users in India according to the Global Adult Tobacco Survey 2016–2017, beedis are the most widely consumed tobacco product in South Asian countries. A beedi, also referred to as ‘biri’, is a tiny, thin, hand-rolled tobacco product wrapped in the leaves of native Asian plants called tendu or temburni, and fastened at one or both ends with a vibrant string^3,4^. In many parts of India, beedi rolling is primarily a home-based, unorganized industry where larger beedi makers rely on subcontractors as their main source of labor. In many villages throughout the nation, women rely mostly on this incredibly labor-intensive beedi rolling as their source of income^5-7^. Labor unions estimate that 7–8 million people work in the beedi industry nationwide, including those involved in tendu leaf collection and beedi commerce^4^. With approximately 5.5 million registered beedi workers in India, beedi rolling remains one of the primary occupations for women across the country, including coastal Karnataka^6^.

In order to control working conditions and offer welfare benefits to beedi workers and their families, the government of India has enacted several laws. According to the Beedi and Cigar Workers (Conditions of Employment) Act 1996, ‘No employee shall be forced or permitted to work more than 9 hours in any one day or 48 hours in any one week in any industrial premises, and every employee is eligible for the calendar year leave’^8^. Women continue to practice it despite being significantly underpaid due to the convenience of beedi rolling at home and flexible work hours, unlike regular industrial settings. The nature of the work is piece-rate; all raw materials are gathered from approved contractors, and completed goods are delivered for the mandated minimum wage^8^. However, the convenience of employment is often overshadowed by other challenges faced by the beedi rollers, like unregulated workplaces, rising health complications, labor exploitation, and loopholes in regulatory frameworks^4,9^.

In view of the challenges faced by beedi rollers, it is important to calculate the full-time equivalent (FTE) by understanding the nature of the work and ascertaining various parameters involved like working days per year, piece-rate systems, level of expertise, experience, and other allowances. Extending the formula for FTE for beedi rollers is therefore necessary, and a big step in the direction of further reforms in this industry. Articles 17 and 18 of the Framework Convention on Tobacco Control (FCTC) of the World Health Organization (WHO) which address ‘Provision of support for economically viable alternative activities’ and ‘Protection of the environment and the health of persons’, respectively^10^, are in line with this research work. It also marks a step towards accomplishing the Sustainable Development Goal: Promoting sustainable and inclusive economic growth, full and productive employment, and decent work for all. With this background, the present research aims to develop a formula for calculating the extended FTE for beedi workers and a pilot test for applicability among the beedi rollers.

## METHODS

### FTE parameters selection

A multifactorial approach was used to determine the parameters for computing the FTE framework including expert opinion, scoping review of unpublished data available to the authors, and qualitative interviews among beedi rollers^11^. The parameters included workers’ identification (registered or unregistered), average working hours per day and week, number of beedis rolled daily and weekly, number of family members involved in the profession, payment, and leaves taken for personal or health issues, supply of raw materials, average number of rolled beedis rejected because of poor quality, and any benefits they receive outside of pay. A 14-item questionnaire tool was developed using these parameters and was pilot-tested among beedi rollers.

### Face and content validity of FTE parameters

Every FTE parameter underwent a comprehensive evaluation for both face^12^ and content validity^13^.

### Face validity

Face validity aims to subjectively assess the extent to which a tool is likely to measure what is expected. Two female and two male experts were chosen to validate the draft questionnaire; two validated the questionnaire in the regional language (Kannada), and the other two in English. They subjectively evaluated the tool for readability, feasibility, relevance of variables, clarity, and consistency of style and formatting. Each of the four experts confirmed the validity of the questionnaire.

### Content validity

This exercise assesses whether a tool comprehensively covers the entire domain of the concept it aims to measure. The structured questionnaire tool to assess the content relevance was given to another four experts. The content validity index (CVI) was computed for each scale item to obtain the scale content validity index (S-CVI)^14-16^ , as shown in Table 1. Further, an item-level CVI (I-CVI) was computed based on the experts rating each item depending on the item’s relevance on a four-point ordinal scale (1=not relevant, 2=somewhat relevant, 3=quite relevant, and 4=highly relevant). The I-CVI was calculated by dividing the number of experts providing a rating of three or four to an item by the total number of experts reviewing it. In addition, to quantify methodological robustness, a modified Cohen’s kappa (κ) value was ascertained to understand the inter-rater agreement that adjusts for the chance agreement of statements by experts and overcomes the ambiguities of I-CVI.

**Table 1:** Details regarding content validation, I-CVI, and Cohen’s kappa values by experts, 2022–2023

*No.*	*FTE items in the questionnaire*	*Expert-1*	*Expert-2*	*Expert-3*	*Expert-4*	*Agree*	*I-CVI*	*Cohen’s κ*	*Interpretation*
1	Identification through: Pass book and Notebook	4	4	2	4	3	0.75	0.67	Good
2	Apart from beedi rolling, do you do any other work?	4	4	2	4	3	0.75	0.67	Good
3	Since how many years are you rolling beedis?	4	4	2	4	3	0.75	0.67	Good
4	How many days you work in a week?	3	4	2	4	3	0.75	0.67	Good
5	How many hours you work in a day?	3	4	3	4	4	1	1	Excellent
6	How many times you will sit for rolling beedis in a day?	3	4	1	4	3	0.75	0.67	Good
7	How many beedis you roll in a day?	3	4	3	4	4	1	1	Excellent
8	How many of beedis you roll in a week?	4	4	1	4	3	0.75	0.67	Good
9	Is there anyone else in the family who rolls beedis other than you?	4	4	2	4	3	0.75	0.67	Good
10	Please indicate the number of days of leave you took in the previous year	3	4	3	4	4	1	1	Excellent
11	In the previous year, how many days were you unable to work due to a lack of raw materials supplied by the checker or company?	3	4	1	4	3	0.75	0.67	Good
12	On average, how many beedis will be rejected by the checker in a week?	3	4	1	4	3	0.75	0.67	Good
13	How much payment you get for rolling 1000 beedis?	4	4	1	4	3	0.75	0.67	Good
14	How do you receive the payment?	4	4	3	4	4	1	1	Excellent
	**Proportion relevance**	1	1	0.29	1				
	**Scale Content Validity Index (S-CVI /Average)**						0.82		

As per the I-CVI threshold criteria, values >0.79 were considered appropriate; values between 0.70–0.79 as items that require revision, and values <0.70 were to be eliminated. In the FTE questionnaire, I-CVI scores ranged from 0.75 to 1.00 for all items. The items scoring from 0.75 to 0.79 were modified, and other items that scored above 0.79 were retained. Except for item 11, which scored <0.7, it was reconsidered, and the question was modified for appropriateness and relevance.

Scale Content Validity Index (S-CVI) is the proportion of items in an instrument that achieved a rating of 3 or 4 by all the content experts^14-16^. The S-CVI was calculated by averaging the scores of the I-CVIs of each item, and the S-CVI score for the study was 0.8284, which exceeded 0.80, representing excellent content validity.

### Cohen’s kappa statistic

To calculate Cohen’s κ statistic, the probability of chance agreement (Pc) for each item was initially computed using a formula that involves the number of experts (N) and the number of experts who agree that the item was relevant (A). The formula incorporates factorial calculations and considers the probability of chance agreement among the experts. The κ value was calculated by using the difference between the I-CVI and probability of chance agreement (Pc) (observed agreement - expected agreement) divided by the difference between 1 and Pc (1 - expected agreement)^17,18^.

As depicted in Table 1, out of the 14 FTE items, ten scored values of ≥0.75, indicating a substantial agreement among the experts, and four items had a score of 1.00 (excellent), indicating a perfect agreement among the experts. The modified κ scores of each item exhibited good and excellent interrater agreement on item ratings (κ* >0.74). Four experts’ overall ratings demonstrated that the questionnaire’s content was significantly valid and consistent regarding domain representation, relevance, and definition.

### Pilot test

Around 10 beedi rollers from Udupi district of Karnataka were recruited for the pilot study using the convenience sampling method. The eligible beedi rollers were identified, and details regarding the FTE parameters were collected using the validated questionnaire tool after obtaining written informed consent. The pilot data were used to calculate FTE.

### Ethical considerations

The study protocol was approved by the Kasturba Medical College and Kasturba Hospital Institutional Ethics Committee 1 (IEC 1: 402/2022), and participant recruitment was done only after the approval.

## RESULTS

With the beedi rolling occupation mostly practiced as a home-based industry and the rollers being paid on a piece-rate system in India, we developed various formula frameworks accounting for the nature of this occupation. The FTE formulas developed were on three models obtained from previously conducted scoping reviews: workload model, which took into account the number of hours spent on beedi rolling weekly and yearly; productivity model based on the number of beedis rolled per hour; and per day and forecast model estimating the actual number of beedi rollers required to meet the demand at a national level. The pilot data collected among ten beedi rollers on various parameters of the formulae were used to calculate the extended FTE for beedi rollers for all three models. This was, in turn, used in the development of the following framework for classifying the FTE into the following categories of workload: underloaded (<0.95), normal (0.95–1.05), and overloaded (>1.05).

### FTE calculation based on the productivity model

Regarding beedi rolling occupation, productivity means the number of beedis rolled per hour and the formula developed calculates FTE as follows:


$$\text{Extended FTE per year}=\frac{\left(\text{Beedis rolled per hour}\times \text{Hours worked per day}\right)\times \text{Effective working days per year}}{\left\{\text{Average beedis rolled per day}\times \left[\left(\text{Days in a year-Sundays in a year}\right)-\left(\text{No. of government holidays + Calendar year leave with wages}\right)\right]\right\}}$$

To find the beedi rolled per hour, the total beedi rolled in a week was divided by the product of the number of days worked in that week and the hours worked per day as per the pilot data. The effective working days in a year were calculated by subtracting off-days and standby days from the total days in a year (Supplementary file Method 1A). The pilot study using the framework based on productivity (per hour beedi rolled) estimated that the beedi roller average of the E-FTE index is 0.67, thus reporting underload (Supplementary file Table 1).

Another way to assess extended full-time equivalent (E-FTE) for a year was through actual beedis rolled daily while keeping the remaining parameters the same as those used in the calculation for beedis rolled hourly (Supplementary file Method 1B). Further, the pilot study results based on this formula indicate that beedi rollers are underloaded with an average E-FTE index of 0.54. (Supplementary file Table 2).

### FTE calculation based on the workload model

This model was based on the number of hours worked weekly, and the formula developed calculates FTE as follows:


$$\text{Extended FTE per week}=\frac{\left(\text{Hours worked per day}\times \text{Number of days employed per week}\right)}{\text{Standard hours of work per day}\times \text{Standard working days per week}}$$

The FTE formula developed, considering the workload for the week and year, has been elaborated in Supplementary file Methods 2A and 2B. This approach provided a measure of weekly productivity relative to typical working standards. Further, the pilot study results using the framework based on workload (using weekly workload data) had an average E-FTE index of 1.03, indicating the workload among beedi rollers as normal, while E-FTE calculated using annual workload data shows that the beedi rollers had average index of 0.88, indicating beedi rollers are underload (Supplementary file Tables 3 and 4).

### FTE calculation based on the forecasting model

This model (Supplementary file Method 3) calculates the number of workers needed to roll the specified number of beedis in a given year using the formula:


$$\text{Actual beedi rollers required}=\frac{\text{Annual beedis rolled in India}}{\left(\text{Beedis rolled per hour}\times \text{Hours worked per day}\right)\times \text{Effective working days per year}}$$

This method used qualitative data (beedis rolled per hour) and pilot data (total beedis rolled annually) to measure overall productivity, assuming that the registered beedi rollers worked according to government norms (Supplementary file Table 5). The projected E-FTE for beedi rollers implies that if beedi rollers are operating at an optimal level, we would require fewer beedi rollers compared to the current number in the country. However, if beedi rollers work below the expected standards, the number of beedi rollers increases significantly.

## DISCUSSION

This study describes the development and validation process of formulae framed to estimate the FTE extended for the beedi rolling occupation. These formulae were also tested on beedi rollers through a short pilot study. Data were collected through a structured questionnaire and validated using I-CVI, S-CVI, and multi-rater κ statistics. The parameters changed regarding the inclusion and exclusion of items and their modification. The various FTE formulae based on productivity, workload, and forecast methods were developed and tested on the beedi rollers through a pilot study. The present study is the first to calculate and test the FTE formula for beedi rolling occupation in India.

Based on the pilot data, the extended FTE calculated for beedi rolling occupation with both productivity and workload methods indicated that 60–70% of the rollers are underloaded, implying that the workload of the beedi rollers is below the optimum. A study conducted in a North Indian State found that an average beedi roller could roll 400–600 beedis a day^19^, while a study from a neighboring district in Karnataka revealed that a roller worked for 4–6 hours a day rolling 460–700 beedis per day^5^. In line with these studies, our study also considered rolling approximately 100 beedis per hour as the norm in calculating the extended FTE.

The forecasting model showed that if beedi rollers work less than the standard practice, the required number of beedi rollers drastically increases. However, considering that all the ways of FTE calculation point towards fewer beedi rollers required for the same production output, the way forward must include exploring alternate livelihood options for the beedi rollers. This is also supported by a study conducted in India, which showed that direct employment in the beedi industry has declined over the last decade, which was consistent for both men and women^1^.

A study done in Bangladesh estimated the total FTE employment in the beedi industry. The study considered a 65% utilization rate of the full-time capacity of a regular employee working on the factory premises and a 26% utilization rate of a contract worker’s full-time capacity, accounting for actual production at 33% of potential output^20^. Though this study calculated FTE only for factory-based workers, the results are in line with the present study in terms of loss of full-time jobs with declining beedi production, emphasizing the need for alternate livelihood for these employees. In recent years, various research studies have tried to explore willingness to move towards an alternate livelihood and have found positive responses from them, given the right opportunities and training^6,21^.

In addition to being the first of its kind, the present study tried to calculate FTE for home-based beedi workers, unlike the previous study, which estimated FTE only for factory-based workers^20^. The study developed a formula for various models based on the nature of beedi rolling occupation, considering the holidays, standby days, paid leaves, number of beedis produced per hour, per day, per week, and per year, as well as number of hours worked per day by the workers. These formulae allowed us to comprehensively calculate FTE with most considerations, even when the wage payment systems and working systems were different. We found that the productivity-based formulae, especially using per hour beedi rolled as a parameter, were the most suitable for calculating FTE in our study based on the fluid working hours, the weekly payment for the beedis based on the number of beedis rolled and the holidays and standby days as well. Other formulae based on workload and forecasting lacked one or the other parameters for consideration and, therefore, were less suitable for calculating FTE.

### Limitations

The method of wage payment for the unorganized sector of beedi rollers in our study exhibits regional specificity, highlighting the importance of customized approaches to labor compensations that suit local contexts and may vary in applicability across different regions. The inclusion of a household-helping hand as a parameter in the FTE calculation is omitted due to its variability across most workers.

## CONCLUSIONS

The current study offers a pilot-tested formulae framework to support beedi roller reforms, debunking misconceptions about their working conditions and creating avenues for challenging claims made by tobacco companies. As beedi rollers remuneration is based on a piece-rate system, i.e. the number of beedis rolled once a week and not on the duration/ time taken for beedi rolling, we conclude that the productivity-based model is most suitable for the beedi rolling occupation.

## Supplementary Material



## Data Availability

The data supporting this research can be found in the Supplementary file. Please refer to the Supplementary file for details regarding the different frameworks for calculating E-FTE for beedi rollers and results of the pilot study for each of the frameworks.
