# Supplementary material for: Development of an extended full-time equivalent framework: A workforce metric for the beedi rolling industry in India
Source: Tob Induc Dis. 2025 Jun 5;23:10.18332/tid/203508. doi: 10.18332/tid/203508 (PMC12142004; doi:10.18332/tid/203508)
Supplement: Supplementary file 1 [file TID-23-75-s1.pdf]

Table 1:

Productivity-based Extended-FTE (using per hour beedi rolled) calculated for beedi rollers of a district of coastal Karnataka, A cross-sectional study, 2022-23.

Beedi rollers	1	2	3	4	5	6	7	8	9	10	Average
**Hours worked per day**	7	7	7	4	5	6	8	4	4	6	**5.8**
**Beedi rolled per hour**	112.2	81.6	30.6	71.4	42.8	142.9	107.1	53.6	35.7	47.6	**72.5**
**FTE**	1.22	0.87	0.34	0.45	0.34	1.25	1.33	0.31	0.20	0.44	**0.67**
**FTE index**	Overload	Underload	Underload	Underload	Underload	Overload	Overload	Underload	Underload	Underload	**Underload**

Table 2:

Productivity-based Extended-FTE (using per day beedi rolled) calculated for beedi rollers of a district of coastal Karnataka, A cross-sectional study, 2022-23.

**Beedi rollers**	1	2	3	4	5	6	7	8	9	10	**Average**
**Hours worked per day**	7	7	7	4	5	6	8	4	4	6	**5.8**
**Beedi rolled per hour**	112.24	81.63	30.61	71.42	42.85	142.85	107.14	53.57	35.71	47.61	**72.56**
**FTE**	0.97	0.69	0.27	0.36	0.27	1.00	1.06	0.25	0.16	0.35	**0.54**
**FTE index**	Normal	Underload	Underload	Underload	Underload	Normal	Overload	Underload	Underload	Underload	**Underload**

Table 3:

Workload-based Extended FTE (using weekly workload data) calculated for beedi rollers of a district of coastal Karnataka, A cross-sectional study, 2022-23.

Beedi rollers	1	2	3	4	5	6	7	8	9	10	Average
**Hours worked per day**	7	7	7	4	5	6	8	4	4	6	**5.8**
**Number of days worked per week**	7	7	7	7	7	7	7	7	7	7	**7**
**FTE**	1.02	1.02	1.02	0.58	0.72	0.87	1.16	0.58	0.58	0.87	**1.03**
**FTE index**	Normal	Normal	Normal	Underload	Underload	Underload	Overload	Underload	Underload	Underload	**Normal**

Table 4:

Workload-based Extended FTE (using annual workload data) calculated for beedi rollers of a district of coastal Karnataka, A cross-sectional study, 2022-23.

**Beedi rollers**	1	2	3	4	5	6	7	8	9	10	**Average**
**Hours worked per day**	7	7	7	4	5	6	8	4	4	6	**5.8**
**Effective working day**	357	350	365	363	365	335	358	335	335	358	**357**
**FTE**	1.08	1.06	1.11	0.63	0.79	0.87	1.24	0.58	0.58	0.93	**0.88**
**FTE index**	Overload	Overload	Overload	Underload	Underload	Underload	Overload	Underload	Underload	Underload	**Underload**

Table 5:

Forecast-based calculation using per day beedi rolled from pilot data of a district of coastal Karnataka, A cross-sectional study, 2022-23.

	Government norms	1	2	3	4	5	6	7	8	9	10
**Beedi rolled per year**	1.14393E+12	1.14E+12	1.14E+12	1.14E+12	1.14E+12	1.14E+12	1.14E+12	1.14E+12	1.14E+12	1.14E+12	1.14E+12
**Number of working days**	365	365	365	365	365	365	365	365	365	365	365
**Effective working days**	287	359	365	365	363	365	335	365	335	335	358
**Number of beedi rolled per day**	800	785.7143	564.2857	214.2857	285.7143	214.2857	842.8571	857.1429	214.2857	142.8571	285.7143
**Beedi rollers required to roll 1 trillion beedis**	4982294	4055479	5554044	14625649	11029673	14625649	4051375	3656412	15935409	23903113	11183719

Figure 1

Method 1 A: Productivity-based Extended FTE formulae using per hour beedi rolled by beedi rollers of a district of coastal Karnataka, A cross-sectional study, 2022-23.

$$\textbf{Extended FTE per year}$$

$$= \frac{(\textbf{Beedi rolled per hour} * \textbf{Hours worked per day}) * \textbf{Effective working days in a year}}{\{\textbf{Average beedi rolled per day} * [(\textbf{No of days in a year} - \textbf{No of sundays in a year}) - (\textbf{No of government holiday} + \textbf{Calendar year leave with wages})]\}}$$

- Beedi rolled per hour $= \frac{\text{Beedi rolled in a week}}{\text{Number of days employed in a week} * \text{Hours worked per day}}$
- Effective working days in a year = (No of days in a year- off days – stand by days)
  - Off days = health issues + festivals or function + [(Weekly off) * 52]
  - Weekly off was calculated as number of days in a week subtracted by the number of days employed in a week
  - Standby days were counted as days when there was lack of raw materials
- Pilot Data: Beedi rolled per hour, Hours worked per day, Beedi rolled in a week, Number of days employed in a week
  - Standard Conditions:
  - Average beedi rolled per day.[10]
  - Number of Sundays in a year
  - Number of government holiday in a year
  - Calendar year leave = Number of days allowed to take leave with wages in a year. [10]

Method 1A: Extended FTE calculated based on individual beedi rollers productivity (using per hour beedi rolled data from pilot study)

**Parameter used in Numerator (Beedi Rolled Per Year According to Pilot Data)**	**Parameter used in Denominator (Beedi Rolled Per Year According to previous literature and Government Norms)**
• $Beedirolled\ per\ hour\ = \frac{6000}{7*8} = 107.1429$<br>• $Beedi\ rolled\ in\ a\ week = 6000$<br>• $Number\ of\ days\ employed = 7$<br>• $Hours\ workedper\ day = 8$<br>• $Number\ of\ days = 365$<br>• $Offs\ days = health\ issues + festivals\ or\ function + [(Weekly\ off) * 52] = 0$<br>• $Weekly\ off\ = Nmber\ of\ days\ in\ a\ week - Number\ of\ days\ empolyed\ in\ a\ week = 7 - 7\ = 0$<br>• $Weeks\ in\ a\ year = 52$<br>• $Offs\ days\ (sundays\ in\ a\ year) = 0$<br>• $Standby\ day\ lack\ of\ raw\ materials = 7$	• $Number\ of\ beedis\ rolled\ in\ a\ day = 500 - 1000$ beedi<br>• $Hours\ worked\ per\ day = 8$<br>• $Average\ beedi\ rolled\ per\ hour =\ 100$<br>• $No\ of\ sundays\ in\ the\ given\ year = 52$<br>• $Number\ of\ days\ in\ a\ year = 365$<br>• $Offs\ days\ (sundays\ in\ a\ year) = 52$<br>• $Government\ holiday = 14$<br>• $Calendar\ year\ leave\ with\ wages = 12$
$Extended\ FTE\ per\ year = \frac{(\ 8\ *\ 107.1429) * (365 - 0 - 7)}{(1000) * [(365 - 52) - (12 +\ 14)]}$<br><br>$Extended\ FTE\ per\ year = \frac{(\ 857.1432) * (358)}{(1000) * [287]}$	$Extended\ FTE\ per\ year = \frac{306857.27}{28700} = 1.07$

Figure 2:

Method 1 B: Productivity-based Extended FTE formulae using per day beedi rolled by beedi rollers of a district of coastal Karnataka, A cross-sectional study, 2022-23.

$$\textit{Extended FTE per year}$$

$$= \frac{(\textit{Beedi rolled per hour} * \textit{Hours worked per day}) * \textit{Effective working days in a year}}{\{\textit{Average beedi rolled per day} * [(\textit{No of days in a year} - \textit{No of sundays in a year}) - (\textit{No of government holiday} + \textit{Calendar year leave with wages})]\}}$$

- Beedi rolled per hour $= \frac{\text{Beedi rolled in a week}}{\text{Number of days employed in a week} * \text{Hours worked per day}}$
- Effective working days in a year = (No of days in a year- off days – stand by days)
  - Off days = health issues + festivals or function + [(Weekly off) * 52]
  - Weekly off was calculated as number of days in a week subtracted by the number of days employed in a week
  - Standby days were counted as days when there was lack of raw materials
- Pilot Data: Beedi rolled per hour, Hours worked per day, Beedi rolled in a week, Number of days employed in a week
  - Standard Conditions:
  - Average beedi rolled per day.[10]
  - Number of Sundays in a year
  - Number of government holiday in a year
  - Calendar year leave = Number of days allowed to take leave with wages in a year. [10]

Method 1B: Extended FTE calculated based on individual beedi rollers productivity (using per day beedi rolled data from pilot study)

**Parameter used in Numerator (Beedi Rolled Per Year According to Pilot Data)**	**Parameter used in Denominator (Beedi Rolled Per Year According to previous literature and Government Norms)**
• $Beedirolled\ per\ hour\ = \frac{6000}{7*8} = 107.1429$<br>• $Beedi\ rolled\ in\ a\ week = 6000$<br>• $Number\ of\ days\ employed = 7$<br>• $Hours\ workedper\ day = 8$<br>• $Number\ of\ days = 365$<br>• $Offs\ days = health\ issues + festivals\ or\ function + [(Weekly\ off) * 52] = 0$<br>• $Weekly\ off\ = Nmber\ of\ days\ in\ a\ week - Number\ of\ days\ empolyed\ in\ a\ week = 7 - 7\ = 0$<br>• $Weeks\ in\ a\ year = 52$<br>• $Offs\ days\ (sundays\ in\ a\ year) = 0$<br>• $Standby\ day\ lack\ of\ raw\ materials = 7$	• $Number\ of\ beedis\ rolled\ in\ a\ day = 500 - 1000$ beedi<br>• $Hours\ worked\ per\ day = 8$<br>• $Average\ beedi\ rolled\ per\ hour =\ 100$<br>• $No\ of\ sundays\ in\ the\ given\ year = 52$<br>• $Number\ of\ days\ in\ a\ year = 365$<br>• $Offs\ days\ (sundays\ in\ a\ year) = 52$<br>• $Government\ holiday = 14$<br>• $Calendar\ year\ leave\ with\ wages = 12$
$Extended\ FTE\ per\ year = \frac{(8\ *\ 107.1429) * (365 - 0 - 7)}{(1000) * [(365 - 52) - (12 +\ 14)]}$<br><br>$Extended\ FTE\ per\ year = \frac{(857.1432) * (358)}{(1000) * [287]}$	$Extended\ FTE\ per\ year = \frac{306857.27}{28700} = 1.07$

Figure 3:

Method 2 A: Workload based Extended FTE formulae using weekly workload of beedi rollers in a district of coastal Karnataka, A cross-sectional study, 2022-23.

$$\boldsymbol{Extended\ FTE\ per\ week} = \frac{(\boldsymbol{Hours\ worked\ per\ day} * \boldsymbol{Number\ of\ days\ employed\ in\ a\ week})}{\boldsymbol{Standard\ hours\ of\ work\ per\ day} * \boldsymbol{Standard\ working\ days\ in\ a\ week}}$$

- Pilot Data: Hours worked per day, Number of days employed in a week
- Standard Conditions:
  - Standard Hours worked per day = 8
  - Expected number of working days in a week

Method 2 A: Extended FTE calculated based on individual beedi rollers working days in a given week, data from pilot study)

**Parameter used in Numerator (Beedi Rolled Per Year According to Pilot Data)**<br>$Number\ of\ days\ employed = 7$<br>• $Hours\ workedper\ day = 8$	**Parameter used in Denominator (Beedi Rolled Per Year According to Government Norms)**<br>• Standard $Number\ of\ days\ employed = 6$<br>• Standard $Hours\ worked\ per\ day = 8$
$Extended\ FTE\ per\ week = \frac{(8 * 7)}{(8 * 6)}$	$Extended\ FTE\ per\ week = \frac{56}{48} = 1.17$

Figure 4:

Method 2 B: Workload-based Extended FTE formulae calculated according to the annual workload of beedi rollers in a district of coastal Karnataka, A cross-sectional study, 2022-23.

$$\boldsymbol{Extended\ FTE\ per\ year} = \frac{(\boldsymbol{Hours\ worked\ per\ day} * \boldsymbol{Effective\ working\ days\ per\ year})}{\boldsymbol{Standard\ working\ hours} * \boldsymbol{Expected\ number\ of\ working\ days\ in\ a\ year}}$$

- Effective working days in a year = (No of days in a year-off days-stand by days)
  - Off days = health issues + festivals or function + [(Weekly off) *52]
  - Weekly off was calculated as number of days in a week subtracted by the number of days employed in a week
  - Standby days were counted as days when there was lack of raw materials

- $$\boldsymbol{Expected\ number\ of\ working\ days\ in\ a\ year} = (\boldsymbol{No\ of\ days\ in\ a\ year} - \boldsymbol{No\ of\ sundays\ in\ a\ year}) - (\boldsymbol{No\ of\ government\ holiday} + \boldsymbol{Calendar\ year\ leave\ with\ wages})]\}$$

- Pilot Data: Hours worked per day, Effective working days in a year
- Standard Conditions:
  - Standard working hours
  - Expected number of working days in a year

Method 2B: Extended FTE calculated according to annual workload of beedi rollers in a district of coastal Karnataka, A cross-sectional study, 2022-23.

**Parameter used in Numerator (Beedi Rolled Per Year According to Pilot Data)**	**Parameter used in Denominator (Beedi Rolled Per Year According to Government Norms)**
• $Hours\ workedper\ day = 8$<br>• $Number\ of\ days = 365$<br>• $Offs\ days = health\ issues + festivals\ or\ function + [(Weekly\ off) * 52] = 0$<br>• $Weekly\ off = Nmber\ of\ days\ in\ a\ week - Number\ of\ days\ empolyed\ in\ a\ week = 7 - 7 = 0$<br>• $Weeks\ in\ a\ year = 52$<br>• $Off\ days\ (sundays\ in\ a\ year) = 0$<br>• $Standby\ day\ lack\ of\ raw\ materials = 7$	• $Hours\ worked\ per\ day = 8$<br>• $Average\ beedi\ rolled\ per\ hour = 100$<br>• $No\ of\ sundays\ in\ the\ given\ year = 52$<br>• $Number\ of\ days\ in\ a\ year = 365$<br>• $Offs\ days\ (sundays\ in\ a\ year) = 52$<br>• $Government\ holiday = 14$<br>• $Calendar\ year\ leave\ with\ wages = 12$
$Extended\ FTE\ per\ year = \frac{(8) * (365 - 0 - 7)}{(8) * [(365 - 52) - (12 + 14)]}$<br>$Extended\ FTE\ per\ year = \frac{(8) * (358)}{(8) * [287]}$	$Extended\ FTE\ per\ year = \frac{2864}{2289} = 1.25$

Figure 5:

Method 3: Forecast-based Extended FTE formulae to estimate actual beedi rollers required in a district of coastal Karnataka, A cross-sectional study, 2022-23.

$$\boldsymbol{Actual\ Beedi\ rollers\ required = \frac{Annual\ beedis\ rolled\ in\ india}{(Beedi\ rolled\ per\ hour * \ Hours\ worked\ per\ day\ ) * Effective\ working\ days\ in\ a\ year}}$$

- $Annual\ beedi\ rolled\ in\ india\ = \{[(Average\ beedi\ rolled\ per\ hour\ * \ Hours\ worked\ per\ day) * (No\ of\ days\ in\ a\ year - (weekly\ off) * 52\ - Goverment\ leaves\ - Casual\ leave)] *$ Registered $beedi\ rollers\ in\ india\}$

  ** This gives the number of beedi rolled annually if the registered beedi rollers work according to the government norms.*

- $Beedi\ rolled\ per\ hour\ = \frac{Beedi\ rolled\ in\ a\ week}{Number\ of\ days\ empolyed\ in\ a\ week * Hours\ worked\ per\ day}$
- Pilot data:

$$Effective\ working\ days\ in\ a\ year = (No\ of\ days\ in\ a\ year - off\ days)$$

$$Off\ days = health\ issues + festivals\ or\ function + [(Weekly\ off) * 52]$$

$$Weekly\ off = Nmber\ of\ days\ in\ a\ week - Number\ of\ days\ empolyed\ in\ a\ week$$

In this method, we calculated the overall number of beedis rolled by registered beedi rollers in India considering beedi rolled per hour from qualitative data to act as numerator and in the denominator, we used the pilot data to calculate the total number of beedis rolled in a year.

<table>
<tr>
<td>

- $Hours\ required\ per\ day = 8$
- $Beedi\ rolled\ per\ hour = 100$
- $Number\ of\ days = 365$
- $Offs\ days\ (sundays\ in\ a\ year) = 52$
- $Government\ holiday = 14$
- $Casual\ leave = 12$
- $Registered\ beedi\ rolles\ in\ India = 49{,}82{,}294$

</td>
<td colspan="2">

$$\boldsymbol{Annual\ beedi\ rolled\ in\ india} = \{[(800) * (365 - 52 - 12 - 14)] *\ 49{,}82{,}294\ \}$$

$$\boldsymbol{Annual\ beedi\ rolled\ in\ india} = 229600 *\ 49{,}82{,}294$$

$$\boldsymbol{Annual\ beedi\ rolled\ in\ india} = 1143934702400$$

</td>
</tr>
<tr>
<td colspan="3">

[The total number of beedi's rolled in India has been calculated according to government norms.]

</td>
</tr>
<tr>
<td rowspan="3">

Parameter used in Numerator

- $\boldsymbol{Annual\ beedi\ rolled\ in\ india} = 1143934702400$

$$\boldsymbol{Approximate\ beedi\ rollers} = \frac{(1143934702400)}{(107.1429 * 8\ ) * (365 - 0 - 7)}$$

$$\boldsymbol{Approximate\ beedi\ rollers} = \frac{(1143934702400)}{(857.14) * (358)}$$

</td>
<td colspan="2">

Parameter used in Denominator (Beedi Rolled Per Year According To Government Norms)

</td>
</tr>
<tr>
<td>

- $Beedirolled\ per\ hour\ = \frac{6000}{7*8} = 107.1429$
- $Beedi\ rolled\ in\ a\ week = 6000$
- $Number\ of\ days\ employed = 7$
- $Hours\ workedper\ day = 8$
- $number\ of\ days = 365$
- $Offs\ days = health\ issues + festivals\ or\ function + [(Weekly\ off) * 52] = 0$

</td>
<td>

- $Weekly\ off\ = Nmber\ of\ days\ in\ a\ week - Number\ of\ days\ empolyed\ in\ a\ week = 7 - 7\ = 0$
- $Weeks\ in\ a\ year = 52$
- $Offs\ days\ (sundays\ in\ a\ year) = 0$
- $Standby\ day\ lack\ of\ raw\ materials = 7$

</td>
</tr>
<tr>
<td colspan="2">

$$\boldsymbol{Approximate\ beedi\ rollers = \ 3656412.291}$$

</td>
</tr>
</table>

© 2025 Eshwari K. et al.
